# Data on collagen structures in leather with varying moisture contents from small angle X-ray scattering and three point bend testing

**DOI:** 10.1016/j.dib.2018.10.083

**Published:** 2018-10-26

**Authors:** S.J.R. Kelly, R. Weinkamer, L. Bertinetti, R.L. Edmonds, K.H. Sizeland, H.C. Wells, P. Fratzl, R.G. Haverkamp

**Affiliations:** a School of Engineering and Advanced Technology, Massey University, Palmerston North 4442, New Zealand; b Department of Biomaterials, Max Planck Institute of Colloids and Interfaces, Research Campus Potsdam-Golm, 14424 Potsdam, Germany; c The New Zealand Leather and Shoe Research Association, Palmerston North 4442, New Zealand; d Australian Synchrotron, Clayton, VIC 3168, Australia; e Proteins and Biomaterials, AgResearch, Lincoln 7674, New Zealand

## Abstract

The data presented in this article are related to the research article entitled “Effect of collagen packing and moisture content on leather stiffness” (Kelly et al., 2018). This article describes how moisture content affects collagen packing and leather stiffness. Structural changes were experimentally introduced into ovine leather through biaxial strain during tanning (׳stretch tanning׳). Leather samples produced normally without strain (׳non-stretch tanned׳) and those produced by stretch tanning, were conditioned in a range of relative humidity environments and then analysed by small angle X-ray scattering and three point bend testing. The collagen D-spacing, lateral intermolecular spacing and flexural properties were measured under these varying moisture contents.

**Specifications table**

**Table:** 

Subject area	*Materials Science*
More specific subject area	*Biomaterials*
Type of data	*Graphs and Tables*
How data was acquired	*Biaxial strain, small angle X-ray scattering (SAXS) and three point bend testing*
Data format	*Analyzed*
Experimental factors	*Samples were pre-conditioned in a controlled humidity environment over saturated salt solutions (for synchrotron based SAXS measurements) or into a custom humidity cell (for bench-top SAXS measurements) to achieve 40, 60 and 80% relative humidity. 100% relative humidity was achieved by wetting the samples. 0% relative humidity was achieved by placing the samples in a vacuum chamber during measurements.*
Experimental features	*Small and wide angle X-ray scattering (SAXS/WAXS) beamline at the Australian Synchrotron using an X-ray energy of 18 keV and a sample to detector distance of 3.30 m for SAXS and 0.56 m for WAXS.*
	*Bench-top SAXS II Nanostar using an X-ray energy of 8 keV in the scanning-SAXS configuration with rotating anode X-ray generator, crossed Göbel mirrors and HiStar area detector (Bruker AXS) under vacuum with a sample detector distance of 1.10 m.*
	*Three point bend testing according to ISO 178:2010 was used for flexural measurements.*
Data source location	*Massey University, Palmerston North, New Zealand*
Data accessibility	*All data is provided in this article.*
Related research article	*S. J. R. Kelly, R. Weinkamer, L. Bertinetti, R. L. Edmonds, K. H. Sizeland, H. C. Wells, P. Fratzl and R. G. Haverkamp. Effect of collagen packing and moisture content on leather stiffness. Journal of Mechanical Behavior of Biomedical Materials (2018).*[1]

**Value of the data**

• The data presents changes in structural parameters of collagen, including D-spacing and intermolecular spacing, with both moisture content and tensile strain during tanning and can be used to test theories of the influence of water on collagen structure.
• The data presents changes in mechanical properties of leather, using the bend test, with changes in moisture content and tensile strain during tanning and can be used to test theories of the relationship between mechanical properties and nanostructure.

## 1 Data

The data presented here offers additional measurements of collagen structural parameters and mechanical properties of leather at different sample orientations to that presented in Ref. [1]. Here we present results from SAXS measured flat on to leather surface for samples tanned normally (control) and those tanned under biaxial strain (stretch tanned leather). The data sets are divided into three parts.

**Fig. 1:**
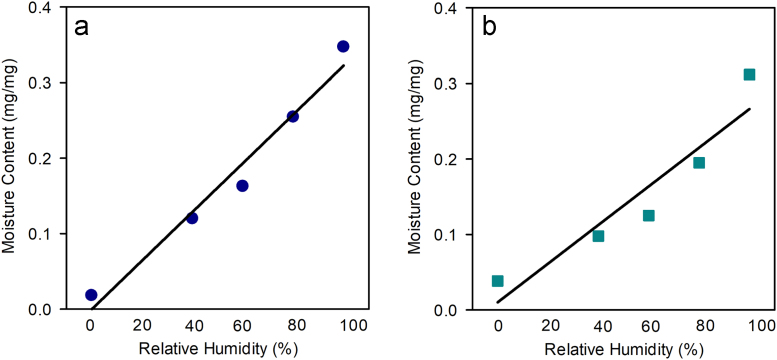
Isothermal gravimetric analysis at various relative humidity environments to determine leather moisture content. Dark blue points are (a) the control leather (slope = 3.2±0.3 µg/mg, *R*^2^ = 0.97); and (b) the stretch tanned leather (slope 2.6±0.5 µg/mg, *R*^2^ = 0.89).

**Fig. 2:**
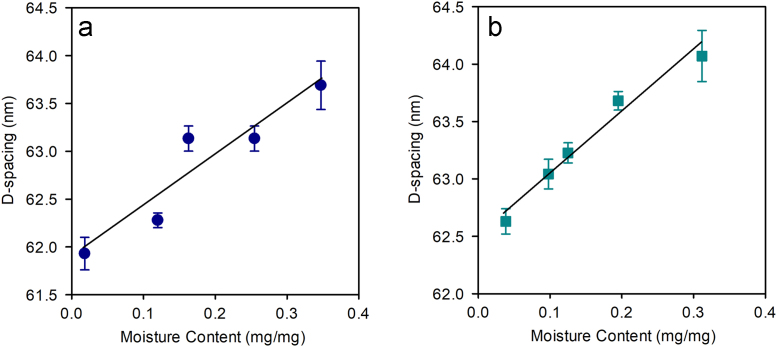
Variations in D-spacing from flat on measurements with moisture content in (a) control leather and (b) stretch tanned leather (slope = 5.11±1.88 *R*^2^ = 0.79, slope = 4.78±0.60 *R*^2^ = 0.97 respectively).

**Fig. 3:**
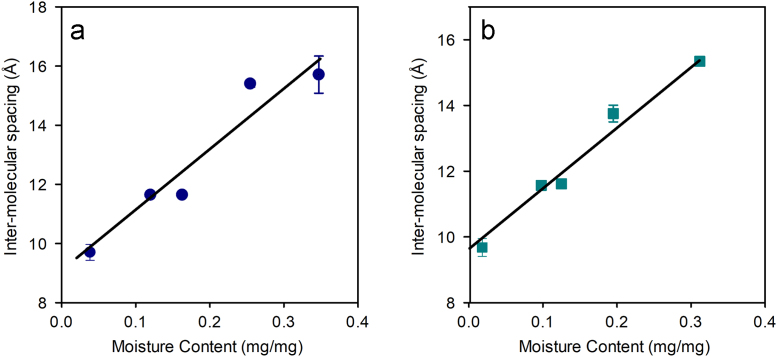
Variations in lateral intermolecular spacing from flat on measurements with moisture content on (a) control leather and (b) stretch tanned leather (slope = 1.60±0.13, 1.35±0.08; *R*^2^ = 0.81, 0.90 respectively). Note the error bars are not visible on some points since the variation within sample repeats was < 0.05.

**Fig. 4:**
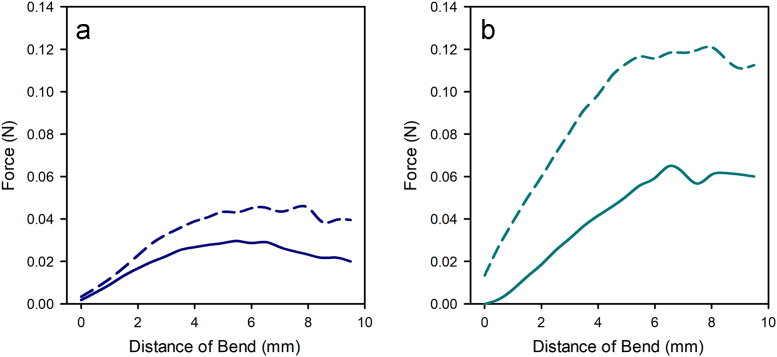
Force deflection curves for (a) control leather and (b) stretch tanned leather where the corium side under tension is represented by the dashed line and the grain side under tension is represented by the solid line.

Part 1 characterizes the thickness (Table 1), apparent density (Table 2) and moisture contents achieved from the various relative humidity environments (Fig. 1) for the two leather types.
Part 2 contains the collagen structural information (D-spacing (Fig. 2 and Table 3) and lateral intermolecular spacing (Fig. 3 and Table 4) of the two leather types at varying moisture.
Part 3 shows the force-deflection curve (Fig. 4) of the two leather types.

### 1.1 Part-1: Characteristics of the two leather types (non-stretch tanned (control) and stretch tanned leather)

See Tables 1 and 2 and Fig. 1.

**Table 1:** Thicknesses of control, non-stretch tanned leather and stretch tanned leather grain and corium layers. Data are presented as the average (standard deviation). *P*-values are from a *t*-test assuming unequal variance between the grain and corium layers of the control and stretch tanned leather and the grain to corium ratio of each leather type. These correspond to Fig. 1 in Kelly et al. (2018) [1].

**Sample**	**Grain thickness (mm)**	**Grain: Corium Ratio**
Control leather (Grain, corium)	0.83 (0.04), 1.41 (0.11)	0.6 (0.1)
Stretch tanned leather (Grain, corium)	0.51 (0.08), 0.43 (0.05)	1.2 (0.1)
*P-value (Grain, corium)*	≪*0.05*, ≪*0.05*	≪*0.05*

**Table 2:** Apparent density of each leather type at ambient conditions.

**Sample**	**Length (m)**	**Width (m)**	**Thickness (m)**	**Volume (m**^**3**^**)**	**Weight (kg)**	**Density (kg/m**^**3**^**)**
Control leather 1	0.01309	0.00991	0.00212	2.75E−07	0.000057	208.7
Control leather 2	0.01338	0.0099	0.00202	2.67E−07	0.000059	220.5
Control leather 3	0.01369	0.00976	0.00205	2.73E−07	0.000059	217.2
Stretch tanned leather 1	0.01175	0.00955	0.00093	1.04E−07	0.000036	345.3
Stretch tanned leather 2	0.01126	0.00977	0.00094	1.04E−07	0.000034	325.7
Stretch tanned leather 3	0.01018	0.00964	0.00092	8.99E−08	0.000031	340.2

### 1.2 Part-2: Structural collagen information determined by small angle X-ray scattering (SAXS) under varying moisture contents

See Tables 3, 4 and 5 and Figs. 2 and 3.

**Table 3:** D-spacing lengths for each leather type after relative humidity conditioning with small angle x-ray scattering measurements made edge on to leather samples (similarly to those reported elsewhere [2]) using bench top SAXS (*top,* Fig. 4 presented in Kelly et al., 2018 [1]) and those made flat on to leather surface using synchrotron based SAXS (*bottom,*Fig. 4). Data are presented as the average (standard deviation). *P*-values are from a *t*-test assuming unequal variances in the D-spacing in the grain vs. corium from edge on measurements, and between the control and stretch tanned leather in the flat on measurements.

**Sample**	**D-spacing after relative humidity conditioning (nm)**				
	**0%**	**40%**	**60%**	**80%**	**100%**
	**Edge on measurements**				
Control, non-stretch tanned leather (Grain, corium)	62.41 (0.33),	62.92 (0.51),	63.50 (0.40),	64.11 (0.16),	64.17 (0.21),
	62.16 (0.39)	62.66 (0.41)	62.71 (0.35)	63.71 (0.16)	63.71 (0.38)
*P-value (Grain vs. corium)*	*0.17*	≪*0.05*	≪*0.05*	≪*0.05*	≪*0.05*
Stretch tanned leather (Grain, corium)	63.03 (0.35),	64.03 (0.44),	64.99 (0.34),	64.58 (0.44),	64.50 (0.50),
	62.58 (0.25)	63.17 (0.15)	63.52 (0.40)	63. 73 (0.35)	64.21 (0.32)
*P-value (Grain vs. corium)*	*0.08*	≪*0.05*	≪*0.05*	≪*0.05*	*0.13*
	**Flat on measurement**				
Control, non-stretch tanned leather	61.93 (0.17)	63.04 (0.13)	63.23 (0.09)	63.68 (0.08)	64.07 (0.22)
Stretch tanned leather	62.63 (0.11)	62.28 (0.08)	63.13 (0.13)	63.13 (0.13)	63.69 (0.25)
*P-value (Control vs. stretched)*	≪*0.05*	≪*0.05*	0.23	≪*0.05*	0.31

**Table 4:** Lateral intermolecular spacing [3] lengths for each leather type after relative conditioning for measurements made edge on to the leather samples using bench top SAXS (*top,* Fig. 5 presented in Kelly et al., 2018 [1]) and those made flat on to the leather surface using synchrotron based SAXS (*bottom,*Fig. 3). Data are presented as averages (standard deviation in parentheses). *P*-values are from a *t*-test assuming unequal variances in the lateral intermolecular spacing of the grain vs. corium from edge on measurements. *P*-values are from a *t*-test assuming unequal variances in the lateral intermolecular spacing in the grain vs. corium from edge on measurements and between the control and stretch tanned leather in the flat on measurements.

**Sample**	**Lateral intermolecular spacing after relative humidity conditioning (Å)**				
	**0%**	**40%**	**60%**	**80%**	**100%**
	**Edge on measurements**				
Control leather *(Grain, corium)*	12.10 (0.10),	12.67 (0.03),	14.64 (0.05),	14.61 (0.14),	16.00 (0.15),
	12.15 (0.09)	12.72 (0.05)	14.51 (0.09)	14.91 (0.04)	15.89 (0.14)
*P-value*	*0.56*	*0.44*	*0.35*	*0.16*	*0.65*
Stretch tanned leather *(Grain, corium)*	12.60 (0.04),	13.01 (0.03),	13.60 (0.06),	14.15 (0.07),	15.79 (0.09),
	12.67 (0.07)	13.18 (0.01)	13.53 (0.11)	14.10 (0.07)	15.71 (0.06)
*P-value*	*0.65*	*0.14*	*0.65*	*0.67*	*0.52*
	**Flat on measurement**				
Control leather	9.68 (0.26)	11.57 (0.03)	11.61 (0.02)	13.76 (0.24)	15.34 (0.05)
Stretch tanned leather	9.70 (0.26)	11.64 (0.02)	11.64 (0.03)	15.40 (0.10)	15.71 (0.59)
*P-value (Control vs. stretched)*	*0.90*	≪*0.05*	≪*0.05*	≪*0.05*	*0.12*

**Table 5:** Collagen fibril structural parameters characterize the collagen fibril structure when dry and wet, with rate of change in structure as water is added to the structure for measurements made edge on to the leather surface using bench top SAXS (*top*), and with measurements made flat on to the leather surface using synchrotron based SAXS (*bottom*). Data are presented as averages (standard deviation in parentheses).

**Sample**	**Parameter**	**Dry**^a^	**Wet**^b^	**Rate of change**	***∆* (dry to wet)**
	**Edge on measurements**				
Control leather *(Grain)*	D-spacing (nm)	62.38 (0.21)	64.17 (0.21)	5.79 (0.96)	2.9%
	Lateral intermolecular spacing (Å)	11.86 (0.53)	16.00 (0.15)	11.89 (2.47)	34.9%
Control leather *(Corium)*	D-spacing (nm)	62.04 (0.20)	63.71 (0.38)	5.24 (0.94)	2.7%
	Lateral intermolecular spacing (Å)	11.86 (0.53)	15.89 (0.14)	11.89 (2.47)	34.0%
Stretch tanned leather *(Grain)*	D-spacing (nm)	63.27 (0.37)	64.99 (0.50)	6.27 (2.03)	2.7%
	Lateral intermolecular spacing (Å)	12.02 (0.15)	15.79 (0.09)	11.79 (0.84)	31.4%
Stretch tanned leather *(Corium)*	D-spacing (nm)	62.58 (0.17)	64.21 (0.32)	5.60 (0.96)	2.6%
	Lateral intermolecular spacing Å)	12.14 (0.13)	15.71 (0.06)	11.10 (0.71)	29.4%
	**Flat on measurements**				
Control leather	D-spacing (nm)	61.93 (0.17)	63.69 (0.25)	5.11 (1.89)	2.8%
	Lateral intermolecular spacing (Å)	9.68 (0.26)	15.69 (0.15)	1.60 (0.13)	63.3%
Stretch tanned leather	D-spacing (nm)	62.63 (0.11)	64.07 (0.22)	4.78 (0.60)	2.3%
	Lateral intermolecular spacing (Å)	9.70 (0.26)	15.43 (0.02)	1.35 (0.08)	59.1%

a Dry represents measurement at 0% relative humidity measurements (under vacuum).

b Wet represents measurement at 100% relative humidity measurements (soaked in water). Dry measurements from the Synchrotron flat on measurements were interpolated from measurements at 40%, 60%, 80% and 100% relative humidity points.

### 1.3 Part-3: Force deflection curves determined by three point bend tests for the two leather types, showing the increased stiffness of the stretch tanned leather

See Fig. 4.

## 2 Experimental design, materials, and methods

Leather was prepared from ovine pelts. The first stages consisted of conventional lime sulfide paint, followed by neutralising and washing, then treatment with a commercial bate enzyme (Tanzyme) and pickling in 20% NaCl and 2% H_2_SO_4_. After this stage some samples were subjected to biaxial tension during the remaining of tanning (stretch tanned) and others were tanned without tension (control). The tension applied resulted in a 3% strain parallel to the backbone of the animal and 15% in the perpendicular direction. The remaining tanning process consisted of degreasing, pretanning with 2% oxizolidine, neutralising and washing, then tanning with 5% chrome sulfate solution and retanned with 2% Tanicor (synthetic tanning agent). The final steps were treatment with 6% fatliquor solution and 0.5% formic acid. At this point the skins under tension were released from tension, washed and dried.

Moisture control was achieved by a controlled humidity cell for *in situ* SAXS measurements on the benchtop NanoStar device) and via pre-treatment in controlled humidity environments using saturated salt solutions for SAXS measurements at the Australian Synchrotron SAXS beamline. The saturated salt controlled humidity environments were also used to condition samples prior to the three-point bend tests.

Diffraction patterns recorded on the SAXS II Nanostar were in the scanning-SAXS configuration with rotating anode Cu Kα X-ray generator, crossed Göbel mirrors and HiStar area detector (Bruker AXS) and an X-ray energy of 8 keV and a sample to detector distance of 1.10 m. For the Australian Synchrotron SAXS measurements an X-ray energy of 18 keV was used with a sample to detector distance of 3.30 m for measurements in the low *Q*-range (Fig. 1(b)) and 0.56 m for the high *Q*-range (Fig. 1(a)) with a Pilatus 1 M detector. The exposure time for diffraction patterns was in the range of 1–5 seconds in gapless mode.

Three point bending tests were performed according to ISO 178:2010 using an Instron 4467 instrument (ITW, Massachusetts, USA) to record the force defection curves.

Processing of the raw SAXS data from the Nanostar used the DPDAK software package [4] while for the Australian Synchrotron data scatterBrain software was used.
